# Supplementary material for: Dynalign II: common secondary structure prediction for RNA homologs with domain insertions
Source: Nucleic Acids Res. 2014 Nov 21;42(22):13939–48. doi: 10.1093/nar/gku1172 (PMC4267632; doi:10.1093/nar/gku1172)
Supplement: SUPPLEMENTARY DATA [file supp_gku1172_nar-02021-z-2014-File012.zip › manual/javadoc/RNAstructure_java_drawing/source/proxy/package-tree.html]

RNAstructure\_java\_drawing.source.proxy Class Hierarchy


JavaScript is disabled on your browser.


- Overview
- Package
- Class
- Tree
- Index
- Help

- Prev
- Next

- Frames
- No Frames

- All Classes

# Hierarchy For Package RNAstructure\_java\_drawing.source.proxy

Package Hierarchies:

- All Packages

## Class Hierarchy

- java.lang.Object
  - RNAstructure\_java\_drawing.source.proxy.DotPlotBackend
  - RNAstructure\_java\_drawing.source.proxy.DotPlotBackendProxyJNI
  - RNAstructure\_java\_drawing.source.proxy.StructureBackend
  - RNAstructure\_java\_drawing.source.proxy.StructureBackendProxyJNI

- Overview
- Package
- Class
- Tree
- Index
- Help

- Prev
- Next

- Frames
- No Frames

- All Classes
